# Performance Level and Cortical Atrophy Modulate the Neural Response to Increasing Working Memory Load in Younger and Older Adults

**DOI:** 10.3389/fnagi.2018.00265

**Published:** 2018-09-11

**Authors:** Eva Bauer, Gebhard Sammer, Max Toepper

**Affiliations:** ^1^Cognitive Neuroscience at the Centre for Psychiatry, University of Giessen, Giessen, Germany; ^2^Department of Psychology, University of Giessen, Giessen, Germany; ^3^Bender Institute of Neuroimaging, University of Giessen, Giessen, Germany; ^4^Research Division, Department of Psychiatry and Psychotherapy, Evangelisches Klinikum Bethel, Bielefeld, Germany; ^5^Division of Geriatric Psychiatry, Department of Psychiatry and Psychotherapy, Evangelisches Klinikum Bethel, Bielefeld, Germany

**Keywords:** aging, working memory, load, gray matter, prefrontal cortex, resources, VBM, fMRI

## Abstract

There is evidence that the neural response to increasing working memory (WM) load is modulated by age and performance level. For a valid interpretation of these effects, however, it is important to understand, whether and how they are related to gray matter atrophy. In the current work, we therefore used functional magnetic resonance imaging (fMRI) and voxel-based morphometry (VBM) to examine the association between age, performance level, spatial WM load-related brain activation and gray matter volume in 18 younger high-performers (YHP), 17 younger low-performers (YLP), 17 older high-performers (OHP), and 18 older low-performers (OLP). In multiple sub regions of the prefrontal cortex (PFC), load-related activation followed a linear trend with increasing activation at increasing load in all experimental groups. Results did not reveal differences between the sub groups. Older adults additionally showed a pattern of increasing activation from low to medium load but stable or even decreasing activation from medium to high load in other sub regions of the PFC (quadratic trend). Quadratic trend related brain activation was higher in older than in younger adults and in OLP compared to OHP. In OLP, quadratic trend related brain activation was negatively correlated with both performance accuracy and prefrontal gray matter volume. The results suggest an efficient upregulation of multiple PFC areas as response to increasing WM load in younger and older adults. Older adults and particularly OLP additionally show dysfunctional response patterns (i.e., enhanced quadratic trend related brain activation compared to younger adults and OHP, respectively) in other PFC clusters being associated with gray matter atrophy.

## Introduction

### Changes in Cognition

Aging is associated with cognitive changes (Park et al., 2002; Hedden and Gabrieli, 2004; Stanziano et al., 2010). However, these changes do not affect all cognitive domains but particularly attentional, mnemonic and executive processes. By contrast, verbal and semantic knowledge are relatively preserved. This is redundant (Park et al., 2002; Singer et al., 2003; Hedden and Gabrieli, 2004). Attentional and executive dysfunctions may manifest in various sub domains as processing speed, cognitive flexibility, focused attention or divided attention (Fozard et al., 1994; Bosworth and Schaie, 1999; O’Sullivan et al., 2001; Park et al., 2002; Singer et al., 2003; Buckner, 2004; Hedden and Gabrieli, 2004; Zimmerman et al., 2006; Kennedy and Raz, 2009; Fjell and Walhovd, 2010; Bauer et al., 2015b). Age-related memory dysfunctions involve short-term memory, episodic long-term memory, and working memory (WM) in particular (Park et al., 2002; Schulze et al., 2011). Decreased WM performance may affect numerous higher-level cognitive operations in everyday-life. In the spatial domain, for example, an age-related WM dysfunction may lead to difficulties in orienting and navigating in unfamiliar environments (Moffat et al., 2006; Cushman et al., 2008).

### Changes in Activation

Decreasing WM performance with advancing age is reflected by functional cerebral changes particularly involving prefrontal brain regions (Reuter-Lorenz et al., 2000; Cabeza, 2002; Davis et al., 2008; Nagel et al., 2009; Rottschy et al., 2012; Clague et al., 2014). The kind of these changes, however, varies across studies so that both quantitative and qualitative differences between older and younger adults were reported. Quantitative differences refer to either increased (‘hyperactivation’) or reduced (‘hypoactivation’) prefrontal cortex activation in older compared to younger adults, whereas qualitative activation differences involve the recruitment of additional brain regions or increased frontal bilaterality in older individuals. Besides differences between the different kinds of activation changes, their interpretation differs across studies. Frontal hypoactivation, for example, was assumed to reflect high neural efficiency or reduced neural resources, whereas frontal hyperactivation or increased bilaterality were interpreted as neural inefficiency, neural compensation or reduced regional specificity (Reuter-Lorenz et al., 2000; Cabeza, 2002; Rypma et al., 2002; Johnson et al., 2004; Park et al., 2004; Rajah and D’Esposito, 2005; Reuter-Lorenz and Lustig, 2005; Zarahn et al., 2007; Davis et al., 2008; Reuter-Lorenz and Cappell, 2008; Holtzer et al., 2009; Nagel et al., 2009, 2011; Bennett and Rypma, 2013).

Reasons for these apparently inconsistent interpretations may be found in differing study designs (different paradigms, methods etc.). Moreover, the meaning of activation differences between older and younger adults is highly dependent from other factors such as performance accuracy. In fact, prefrontal hyperactivation or increased bilaterality in older adults together with equal or higher performance accuracy compared to younger adults were consistently attributed to successful compensation (Cabeza, 2002; Reuter-Lorenz and Lustig, 2005). In this case, an older individual enables more neural resources to achieve the same performance as a younger individual. By contrast, hypoactivation and lower performance accuracy in older compared to younger adults suggests failed compensation due to limited neural resources. Contrary to such compensation theories, efficiency theories assume that age-related prefrontal hyperactivation or increased bilaterality together with lower performance accuracy are signs of neural inefficiency, whereas hypoactivation associated with high performance accuracy may indicate neural efficiency. Overall, there is a need for designs allowing the differentiation between attempted and successful compensation to specify the meaning of functional cerebral changes in the aging brain (Sun et al., 2014).

Another factor modulating age-related activation differences is WM load. In fact, pronounced age effects were found by paradigms in which task demands were experimentally manipulated (Nagel et al., 2009; Toepper et al., 2014; Bauer et al., 2015a). Higher task load requires an enhanced recruitment of neural resources being reflected by increased prefrontal brain activation also referred to as ‘neural upregulation.’ In older individuals, this upregulation is associated with altered prefrontal activation patterns as indicated by both imaging and neurophysiological data (McEvoy et al., 2001; Reuter-Lorenz and Cappell, 2008; Nagel et al., 2009; Cappell et al., 2010; Schneider-Garces et al., 2010; Bennett et al., 2013; Toepper et al., 2014; Bauer et al., 2015a).

### Compensation-Related Utilization of Neural Circuits Hypothesis

The most popular aging model considering both performance accuracy and task load is the Compensation-Related Utilization of Neural Circuits Hypothesis (CRUNCH). The CRUNCH model was published by Reuter-Lorenz and Cappell (2008) and postulates that older adults show unimpaired WM performances at low task load but frontal hyperactivation or increased bilaterality reflecting a recruitment of additional neural resources to compensate for an age-related reduction of WM capacity. At high task demands, by contrast, older adults show poorer performances than younger adults associated with frontal hypoactivation suggesting restricted neural resources in older individuals. The CRUNCH predictions were replicated in numerous studies (Nagel et al., 2009; Cappell et al., 2010; Schneider-Garces et al., 2010; Bennett et al., 2013; Toepper et al., 2014). Toepper and colleagues, for example, used functional magnetic resonance imaging (fMRI) to examine spatial WM related brain activation in 45 healthy volunteers between 20 and 68 years of age (Toepper et al., 2014). WM load was manipulated by varying the length of target sequences. Results revealed increased prefrontal activation in older subjects at low compared to high task load, whereas younger subjects showed the opposite pattern. Compared to younger adults, older adults showed increased activity at low task load and decreased activity at high task load. The results furnish proof for a double dissociation between older and younger adults suggesting that the neural response to increasing task load is impaired in older adults. Since prefrontal activation intensity at low task load was positively correlated with the number of errors, the findings suggest that increased activity at low task load may reflect neural inefficiency rather than compensation.

### Performance Level

Noteworthy, an alternative option to examine the association between WM performance and brain activation is the comparison between high- and low-performers. FMRI results of Bauer and colleagues, for example, indicated that younger and older high-performers showed similar patterns of increasing prefrontal activation with increasing WM load (Bauer et al., 2015a). This pattern was less differentiated in older than in younger high-performing adults (i.e., less sharp increase of activation intensity with increasing load), but still suggested an efficient ‘youth-like’ recruitment of neural resources as response to increasing task load. Younger low-performers also showed increased activation at medium task load, but no further increase at high task load, indicating that neural resources of younger low-performers may have been exhausted earlier compared to those of younger and older high-performers. The poorest performances were shown by older low-performers. Moreover, older low-performers showed no pattern of neural upregulation at higher task load (i.e., no increasing activation intensity with increasing load) suggesting that a resource ceiling may already have been reached at lower task load. Very similar effects were previously reported by Nagel et al. (2009), although a different paradigm, different methods and different load levels were utilized (e.g., parallel vs. serial target presentation, load levels 1-3-7 vs. 4-5-6). Together, both studies confirm the validity of the effects described above Toepper et al. (2016). In addition, however, the results of Nagel and colleagues revealed right dorsolateral prefrontal activation following a quadratic trend in older low-performers. These findings suggest that older low-performers show increased prefrontal brain activation at medium compared to low task load. After that, activation intensity reaches a plateau before it diminishes at high task load. Due to these differing results, one aim of the present work was to specify the patterns of load-related prefrontal activation in low-performing individuals, and older low-performers in particular.

### Changes in Gray Matter

A second aim addressed the association between age, performance level and changes in gray matter. Gray matter alterations in older individuals were reported by several studies and can be reflected by cortical thinning, a decreased brain tissue surface or a reduction of brain volume after the second decade of life (Good et al., 2001; Fleischman et al., 2014; Storsve et al., 2014). Reasons for this decline are a shrinkage of neurons, a reduction of synaptic spines and a reduced number of synapses rather than neuronal loss (Fjell and Walhovd, 2010). Global gray matter volume reaches its maximum in early adulthood and then shows a relative constant reduction until the age of 70 (Good et al., 2001) with an annual atrophy rate of approximately 0.2% (Fox and Schott, 2004). Afterward, however, longitudinal data suggest an accelerated degeneration of 0.3–0.5% per year (Fox and Schott, 2004).

Gray matter volume loss does not affect all brain regions to the same extent. Whereas gray matter volume of parahippocampal, cingulate or occipital brain structures appears to be relatively preserved from age-related atrophy (Raz et al., 1997), gray matter reductions were consistently observed in superior parietal and inferior temporal cortices as well as in hippocampus, insula, striatum, and prefrontal cortex (Raz et al., 1997, 2003, 2005; Resnick et al., 2003; Fjell et al., 2009; Fjell and Walhovd, 2010; Di et al., 2014). Among these brain regions, prefrontal cortex seems to show a specific vulnerability to gray matter atrophy (Raz et al., 1997; Fjell et al., 2009). This atrophy is associated with impaired memory, attention and executive processes indicating the relevance of prefrontal cortex integrity for various cognitive domains (Tisserand et al., 2004; Zimmerman et al., 2006; Cardenas et al., 2011; Kaup et al., 2011; Bauer et al., 2015b). WM represents an interface between memory, attention and executive functioning. Consequently, numerous studies reported an association between prefrontal brain volume and WM performance as well (Goldstein et al., 2011; Kaup et al., 2011).

### Rationale of the Study

Taken together, there is much evidence regarding age-related changes in cognition, brain structure and cerebral activation. However, there is sparse evidence for how these different factors are related to each other. Specifically, it remains unclear whether altered load-related prefrontal activation patterns in older adults are linked to age-related prefrontal gray matter atrophy or whether these changes are independent processes manifesting at higher ages.

In the current experiment, we therefore used fMRI and voxel-based morphometry (VBM) to examine the association between performance level, spatial WM load-related brain activation and prefrontal gray matter volume in different age cohorts.

Based on the previous theoretical considerations, younger age and higher performance level should be associated with an efficient upregulation of prefrontal cortex activation as neural response to increasing WM load. Particularly in younger individuals and older high-performers, we therefore expect an increase of prefrontal activation from the lowest to the highest load level (positive linear trend) as quantified by fMRI. Specifically, we expect increased linear trend related activation in younger compared to older adults and in older high-performers compared to older low-performers. In older low-performers, by contrast, we particularly expect increasing load-related activation from low to medium task load but stable or decreasing load-related activation from medium to high task load as indicated by quadratic trends. These quadratic trends probably reflect dysfunctional neural responses. Consequently, older low-performers should show increased quadratic trend related activation compared to older high-performers. Moreover, we expect increased quadratic trend related activation in older compared to younger adults.

Regarding structural differences, we expect lower gray matter volume in older compared to younger individuals and in older low-performers compared to older high-performers in dorsolateral and ventrolateral prefrontal brain regions as quantified by VBM.

Finally, the extent of quadratic trend related activation should be negatively correlated with prefrontal gray matter volume and performance accuracy in older low-performers, indicating a neural dysfunction being associated with structural atrophy.

## Materials and Methods

### Participants

The study included 70 healthy volunteers between 20 and 80 years of age. Participants were divided into two groups of 35 younger and 35 older individuals. To analyze the impact of performance level, both age groups were further subdivided into high-performers and low-performers by median split (errors in the experimental paradigm), eventually resulting in four experimental groups (**Table 1**): younger high-performers (YHP), younger low-performers (YLP), older high-performers (OHP), and older low-performers (OLP). Sample size was determined by an *a priori* power analysis using G^∗^Power 3.1 (*N* = 68; α = 0.05, effect size = 0.3, number of groups = 4, number of measurements = 4).

**Table 1:** Sample characteristics.

	YHP	YLP	OHP	OLP
*N*	18	17	17	18
Sex (female/male)	9/9	11/6	7/10	10/8
Mean age/SD	27.61/5.02	26.71/5.21	57.88/5.38	64.06/9.26
Minimum age	20	20	50	50
Maximum age	35	35	69	80
School education/SD	12.72/.75	12.47/1.01	11.47/1.63	10.78/2.05
Minimum school education	10	10	8	8
Maximum school education	13	13	13	13
MoCA score/SD	28.83/.62	27.47/2.45	27.18/2.43	26.67/2.4
MWT score/SD	32.5/2.31	30.06/2.41	31.53/4.06	32.22/2.56

*Age and school education are given in years. YHP, younger high-performers; YLP, younger low-performers; OHP, older high-performers; OLP, older low-performers; SD, standard deviation; MoCA, Montreal Cognitive Assessment; MWT, multiple choice vocabulary test.*

All participants had normal or corrected-to-normal vision. None of the participants had a documented diagnosis of neurological or psychiatric disease in the past. Global cognitive deficits were excluded by the Montreal Cognitive Assessment (MoCA) (Nasreddine et al., 2005). Participants were recruited by local advertising and provided a written declaration of consent prior to study start. All participants received an expense allowance of 8 € per hour. The study obtained ethical approval by the Institutional Review Board of the University of Giessen being in accordance with the Declaration of Helsinki. Parts of the sample participated in Bauer et al. (2015a).

Next to the MoCA score, we collected the years of school education and scores in the multiple choice vocabulary test (MWT) (Lehrl et al., 1995) to estimate the level of education. YHP and YLP did not differ with respect to age and school education, but with respect to the MoCA score [*t*(33) = 2.28; *p* = 0.029] and the MWT score [*t*(33) = 3.06; *p* = 0.004]. OHP and OLP did not differ with respect to school education, MoCA score and MWT score, but with respect to age [*t*(33) = -2.39; *p* = 0.023]. However, age distribution in both OHP and OLP was rather bimodal and variances were different (*F* = 3.4, *p* = 0.018). Accordingly, a median test was computed not indicating different medians (OHP = 56; OLP = 60; χ^2^= 1.45, *p* = 0.23).

### Task and Experimental Procedure

**FIGURE 1:**
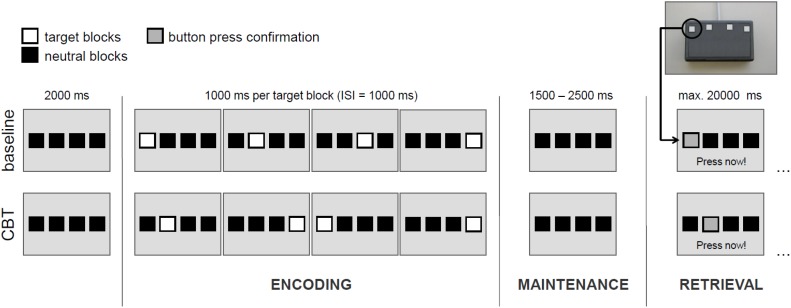
Exemplary illustration of the experimental design for load level 4. CBT, Corsi Block-Tapping test; ISI, inter-stimulus interval. Please note that in the experimental conditions (load levels 4, 5, and 6) one and the same target block may appear more than once within a sequence.

To assess spatial WM, we used a modified version of the Corsi-Block-Tapping test (CBT) (Corsi, 1972; Toepper et al., 2014). The CBT is a multiple item WM task that requires the encoding, maintenance and retrieval of spatial target sequences. WM load can be manipulated by a variation of sequence length. The original (Toepper et al., 2010a,b) and the modified (Toepper et al., 2014) CBT versions reveal nearly identical activation patterns. Basically, four potential target locations are shown on a screen as indicated by four horizontally arranged blocks. Four (load 4), five (load 5) or six (load 6) locations were randomly presented one after another and had to be reproduced by the participant in the correct temporal order afterward. In the baseline condition (load c), all four target locations were presented from left to right (see **Figure 1**). Each participant performed four trials per CBT sequence length and eight baseline trials. The different experimental conditions were counterbalanced with the same sequence for all participants.

Each experimental trial consisted of an encoding phase (in which participants were instructed to learn the presented sequence), a maintenance phase (delay period), and a retrieval phase (during which participants were instructed to reproduce the presented sequence).

The encoding phase started with the onset of the first target block of every sequence and ended after the presentation of the last one. Duration of each target block and inter-stimulus intervals between the blocks were 1000 ms. Hence, in dependence of the respective load level, the encoding phase duration varied between 7000, 9000, and 11.000 ms.

After the encoding phase, the maintenance phase (delay period) started. Duration of the maintenance phase was set to 1500, 2000, or 2500 ms (equally distributed across conditions) in order to improve the event-related sampling quality and efficiency of the design. During the maintenance phase, only the four horizontal blocks were shown and participants were required to keep in mind the sequence presented before.

After the maintenance phase, the retrieval phase started with the instruction “Press now” at the bottom of the screen. During the retrieval phase, participants were required to reproduce the sequence of target blocks presented before in the correct temporal order by performing sequential button presses on a keypad with four horizontally arranged buttons. Each of these four buttons represented the corresponding block on the screen. A direct feedback was given confirming each button press by a change of the of the respective block’s color. The retrieval phase lasted until the last button press (but with a maximum length of 20.000 ms). Overall, each participant performed 20 trials with a duration of about 10 min.

Before the experiment, participants were instructed to memorize the correct locations and temporal order of the presented target blocks. For retrieval, participants were advised to reproduce the presented target sequences by successive button presses and to respond as fast and as accurate as possible. Additionally, participants performed a 2-min series of practice trials on a PC outside the scanner, including two load c and one load 5 trial.

### Stimulus Material

The four horizontally arranged black blocks (RGB 0 0 0) were displayed on a gray background (RGB 163 163 163). Target blocks were displayed in red (RGB 255 0 0). Yellow color after button press indicated the given response (RGB 255 255 0).

### Data Acquisition

A three Tesla Siemens Magnetom Verio Scanner was used for data acquisition. Functional images were recorded using a T2^∗^-weighted echo planar imaging (EPI) sequence (30 slices covering the whole brain; descending order parallel to the AC-PC line +25°; slice thickness = 4 mm; 1 mm gap; TR = 2100 ms; TE = 30 ms; flip angle = 90°; field of view = 192^∗^192 mm; matrix size = 64^∗^64; voxel size = 3^∗^3^∗^4 mm). Via a dual-mirror mounted to the head coil, participants saw the visual stimuli presented on a monitor near the end of the tube. Before the EPI sequence, field map sequences were applied to control for magnetic field inhomogeneities. T1-weighted structural images were assessed with 160 sagittal slices of 1 mm slice thickness using a magnetization prepared rapid gradient echo (MPRage) sequence (TR = 1900 ms; TE = 2.52 ms; flip angle = 9°; field of view = 250 mm; base resolution 256; 176 slices). Time of acquisition in the scanner was approximately 20 min per participant.

### Data Analysis

#### Behavioral Data Analysis

To analyze the impact of load and group on the number of CBT errors, we computed a 4 (CBT load: load c, load 4, load 5, load 6) × 4 (group: YHP, YLP, OHP, and OLP) repeated measures ANOVA including Bonferroni-tests for *post hoc* comparisons. Demographic group differences of interest (age, education, and MoCA score) were analyzed using two-sample *t*-tests. SPSS Statistics 22 was used for behavioral data analyses. Significance level was set to *α* = 0.05, two-tailed.

#### Functional Brain Data Analysis

For the analysis of the MRI data, SPM12 (Statistical Parametric Mapping Software; Wellcome Institute of Neurology at University College, London, United Kingdom)^1^ was used running under MATLAB R2016a (The Mathworks, Natick, MA, United States). To account for the time needed for the magnetic field to become a steady state, the first three images of every EPI-recording session were discarded. Preprocessing of the EPI images included unwarping and realignment to the first volume (b-spline interpolation), slice time correction, normalization to the standard space of the Montreal Neurological Institute (MNI) brain, and smoothing with an isotropic three-dimensional Gaussian kernel with a full-width-at-half-maximum (FWHM) of 9 mm. Data were analyzed using a general linear model (GLM) with four regressors for the encoding phase (load c encoding, load 4 encoding, load 5 encoding, load 6 encoding), one regressor for the maintenance phase, and four regressors for the retrieval phase (load c retrieval, load 4 retrieval, load 5 retrieval, load 6 retrieval). All instead of only correct trials were included into the design to avoid type 1 error due to different trial numbers at the different load levels (Bauer et al., 2015a). Although past work has shown that results do not differ very much, this point is often heavily discussed. In fact, all aging studies are confronted with this problem since both options include advantages and disadvantages. Analyzing only correct trials leads to a different number of analyzed trials in the different experimental groups. Particularly in experimental designs modulating the load level (as in ours), analyzing only correct trials might lead to type 1 error. In the current work (as in many others) the number of correct trials decreased with increasing load but this load-related decrease differed between the different experimental groups. Consequently, a group × load interaction may be the statistical consequence of different trial numbers and not the consequence of activation differences. To avoid this, we decided to include all trials into brain data analyses. However, it must be stated that analyzing all trials might also lead to false positive findings as activation differences may be the result of specific neural activity elicited by incorrect trials. For example, there could be a brain region being more active when responses are incorrect and therefore responds more to increasing load in older than in younger adults. Beside all of these arguments, however, it remains clear that including or excluding incorrect trials refer to substantially different hypotheses.

Previous findings revealed that age-related changes within memory seem to particularly affect the acquisition and early retrieval of new information (Small et al., 1999), which was confirmed for spatial WM retrieval by our working group (Toepper et al., 2014; Bauer et al., 2015a). The current study also focused on WM retrieval. Consequently, only the contrasts related to retrieval were analyzed on the second level. The timing of the regressors followed the timing described in section 2.2. In addition, six movement regressors were modeled. All regressors were convolved with the hemodynamic response function of SPM12. The design matrix was high pass filtered with 128 s.

We built contrasts for positive linear (-3/-1/+1/+3) and quadratic (-1/+1/+1/-1) trends. Theoretically, positive linear trends reflect continuously increasing activation with every higher load level indicating an efficient upregulation of neural activity at increasing load. Quadratic trends, by contrast, reflect an increase of activation from load c to load 4/5 and a decrease from load 4/5 to load 6 suggesting a resource ceiling at load 4/5 (dysfunctional upregulation). Noteworthy, activation patterns that do not strictly follow the described patterns can also reach significance in trend analyses: A positive linear trend, for example, may be characterized by increasing activation from load c to load 4 with no further increase from load 4 to load 5 or 6. This pattern would rather indicate a neural resource ceiling at load level 4 than an efficient upregulation. Similar variations can be observed for quadratic trends and there may even be activation patterns that follow both linear and quadratic functions. Consequently, we plotted mean contrast estimates for the different load levels and experimental groups separately to be able to specify the linear and quadratic trends identified by our analyses.

Neural activation patterns associated with linear and quadratic trends were analyzed using one-sample *t*-tests for each group separately. Data were analyzed at whole-brain level and by a region of interest (ROI) approach, both on the voxel level with a significance threshold of *p* < 0.05 and a family-wise error (FWE) correction for multiple comparisons. Differences between older and younger adults and between OHP and OLP regarding linear and quadratic trend related neural activation were analyzed using two-sample *t*-tests. Again, data were analyzed at whole-brain level and by a ROI approach, both on the voxel level with a significance threshold of *p* < 0.05 and an FWE correction for multiple comparisons.

Based on the theoretical considerations regarding the functional vulnerability of the prefrontal cortex (PFC) in older adults (see Introduction), *a priori* defined ROIs included different dorsolateral and ventrolateral PFC sub regions, including Brodmann areas (BAs) 9 and 46 as well as 44, 45, and 47, respectively. For this, we used the corresponding ROI masks of the automated anatomical labeling atlas (AAL) (Tzourio-Mazoyer et al., 2002), implemented in the WFU PickAtlas (Maldjian et al., 2003).

To be able to draw conclusions about the meaning of quadratic trend patterns for behavioral performance (i.e., ineffectiveness, dysfunctionality), the association between the extent of quadratic trend related brain activation and performance accuracy in OHP and OLP was examined by calculating bivariate Pearson correlations between the contrast estimates in the respective significant peak voxels and the total number of CBT errors. Significance level was set to *α* = 0.05, two-tailed.

#### Structural Brain Data Analysis

##### Data processing

For VBM analyses, all MR structural image data were processed using the CAT12 toolbox^2^ in SPM12^3^ running under MATLAB R2016a (The Mathworks, Natick, MA, United States). Before preprocessing, all structural images were inspected for artifacts and the origin of each image was set at the anterior commissure.

For preprocessing, T1 weighted images initially were spatially registered and segmented into gray matter (GM), white matter (WM) and cerebrospinal fluid (CSF) using the tissue probability maps (TPM) provided by SPM12. This procedure resulted in modulated normalized volumes reflecting the relative differences in regional GM volume. After preprocessing, a quality check was done by visual inspection of the resulting files and by positively checking the sample homogeneity using the implemented tool in CAT12. Finally, data were smoothed with a kernel of 8 mm in SPM12. Total GM volume as well as total intracranial volume (TIV) consisting of GM, WM, and CSF were estimated per subject for statistical analysis.

All GM volume analyses were assessed using the GLM as implemented in SPM12. Gaussian random field theory was applied to estimate the significance of each effect. TIV was used as globals, because it was not orthogonal to the regressors of interest. Sex and years of school education were included as covariates of no interest for all analyses. Absolute threshold for masking was set to 0.00333 (the absolute threshold of 0.1 was divided by 30, as global normalization was applied). GM volume differences between younger and older adults and between OHP and OLP were analyzed at whole-brain level and in different prefrontal ROIs using two-sample *t*-tests with a peak voxel significance threshold of *p* < 0.05 and an FWE correction for multiple comparisons (voxel level). Significant voxels of the whole-brain analysis were labeled using the Adult brain maximum probability map (“Hammersmith atlas”^4^; Hammers et al., 2003; Gousias et al., 2008). To account for age differences between OHP and OLP, age was included as covariate of no interest into the respective analysis. ROI analyses included the same ROIs as the fMRI analyses (see Functional Brain Data Analysis).

#### Relationship Between Functional and Structural Brain Data

In addition, we conducted multiple regression analyses including both functional and structural MRI data. Prefrontal GM volume was included as covariate of interest to analyze the relationship between the extent of the positive linear or quadratic patterns and prefrontal GM volume in the group of older individuals and the sub groups of OHP and OLP. To estimate GM volume in dorsolateral and ventrolateral PFC sub regions, the respective ROIs (BAs 9 and 46, as well as 44, 45 and 47) were coregistered on the preprocessed GM images (smoothed, modulated and warped). Following this, the averaged GM volume of this mask was readout for each subject and included in the statistical analysis described above. Functional ROIs were tested at a significance threshold of *p* < 0.05 at the voxel level with an FWE correction for multiple comparisons.

## Results

### Behavioral Data

**FIGURE 2:**
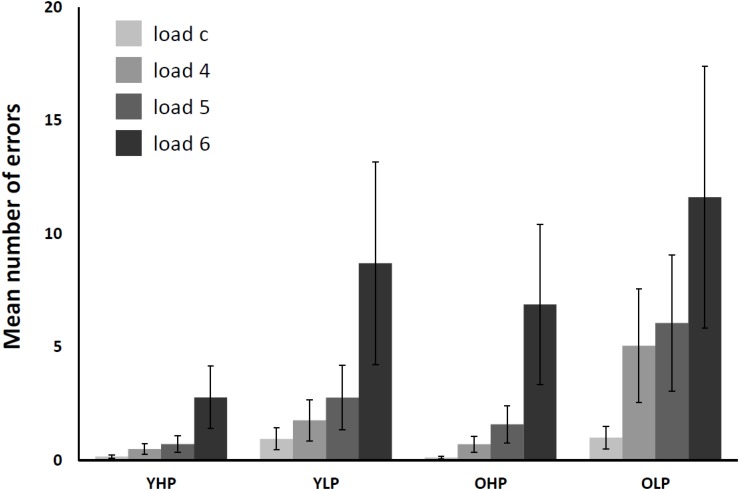
Mean number of total CBT errors in younger high-performers (YHP), younger low-performers (YLP), older high-performers (OHP) and older low-performers (OLP) for all load levels separately (displayed together with standard errors of the mean). Please note that there might be multiple errors in one single trial (i.e., one for each target block).

The 4 (CBT load: load c, load 4, load 5, load 6) × 4 (group: YHP, YLP, OHP, and OLP) repeated measures ANOVA for the number of errors (see **Figure 2**) revealed significant main effects of group [*F*(3,66) = 61,88, *p* < 0.0001] and load [*F*(3,198) = 157,11, *p* < 0.001], as well as a significant group x load interaction effect [*F*(9,198) = 9.64, *p* < 0.001]. *Post hoc* Bonferroni-tests indicated significant differences for all groups and all load levels (*p* < 0.05). The results indicate a disproportionally increasing number of errors with increasing load in all groups. OLP made the most errors, followed by YLP, OHP, and YHP. Moreover, the load-related increase of error rates varied between the groups.

### Functional Brain Data

#### Linear Trend

Results did not reveal significant positive trends at whole-brain level for OHP and OLP. For YHP, positive trend related activity was found within bilateral superior frontal gyrus and left inferior parietal lobule. For YLP, positive trend related activity was found within bilateral superior frontal gyrus (see **Appendix**, **Table A1**).

**FIGURE 3:**
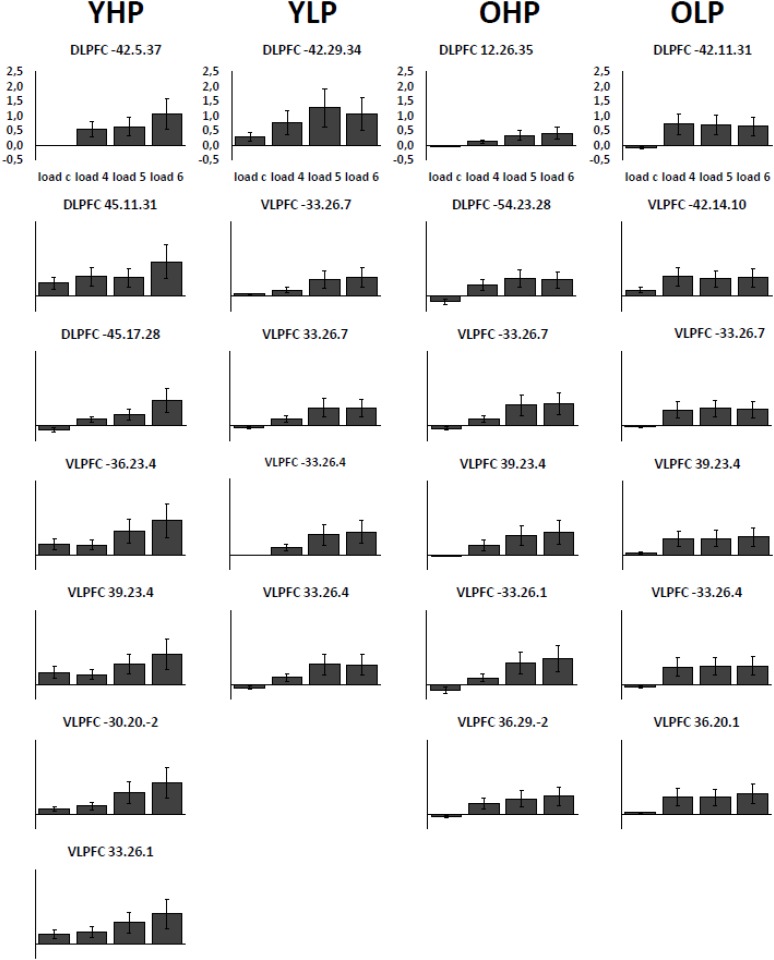
Contrast estimates with the respective standard errors of the mean for the identified regions associated with a positive linear trend. Contrast estimates are plotted for YHP, YLP, OHP and OLP for all load levels separately.

Region of interest analyses revealed significant positive trends for all four groups in different sub regions of the dorsolateral (BAs 9 and 46) and the ventrolateral (BAs 44, 45, 47) PFC. These trends indicate an efficient upregulation of neural activity with increasing task load (**Table 2** and **Figure 3**).

**Table 2:** Positive linear and quadratic trend associated brain activation across the four load levels in different dorsolateral and ventrolateral PFC sub regions (ROI analyses), separately for younger high-performers (YHP), younger low-performers (YLP), older high-performers (OHP), and older low-performers (OLP).

Trend	Group	Region	ROI	Hemisphere	*x*	*y*	*z*	*T*	*p_corr_*
Linear	YHP	DLPFC	BA 9	Left	-42	5	37	5.00	0.011
				Right	45	11	31	4.31	0.040
			BA 46	Left	-45	17	28	3.81	0.032
		VLPFC	BA 45	Left	-36	23	4	4.10	0.017
				Right	39	23	4	4.19	0.015
			BA47	Left	-30	20	-2	5.92	0.001
				Right	33	26	1	6.03	0.001
	YLP	DLPFC	BA 9	Left	-42	29	34	4.55	0.028
		VLPFC	BA 45	Left	-33	26	7	4.00	0.023
				Right	33	26	7	4.72	0.006
			BA 47	Left	-33	26	4	4.45	0.019
				Right	33	26	4	5.57	0.002
	OHP	DLPFC	BA 9	Right	12	26	35	4.41	0.033
			BA 46	Left	-54	23	28	3.75	0.042
		VLPFC	BA 45	Left	-33	26	7	5.17	0.002
				Right	39	23	4	4.21	0.014
			BA 47	Left	-33	26	1	5.55	0.002
				Right	36	29	-2	5.48	0.003
	OLP	DLPFC	BA 9	Left	-42	11	31	4.94	0.005
		VLPFC	BA 44	Left	-42	14	10	3.37	0.045
			BA 45	Left	-33	26	7	5.32	0.001
				Right	39	23	4	4.20	0.004
			BA 47	Left	-33	26	4	5.42	0.002
				Right	36	20	1	5.14	0.004
Quadratic	YHP								
	YLP								
	OHP	DLPFC	BA 9	Left	-42	2	31	5.42	0.005
	OLP	DLPFC	BA 9	Left	-42	11	31	4.98	0.005
			BA 46	Left	-39	35	16	2.12	0.049
				Right	36	35	16	2.24	0.039
		VLPFC	BA 44	Left	-51	14	16	3.49	0.035
			BA 45	Left	-33	26	7	4.37	0.004
				Right	36	26	7	3.74	0.010
			BA 47	Left	-33	26	4	4.33	0.024
				Right	36	26	4	4.08	0.036


*Threshold of *p*_*corr*_ < 0.05 (FWE-corrected according to SPM12). All coordinates (x, y, z) are given in MNI space. DLPFC, dorsolateral prefrontal cortex; VLPFC, ventrolateral prefrontal cortex; ROI, region of interest; BA, Brodmann area; PFC, prefrontal cortex.*

Direct statistical group comparisons indicated no linear trend related activation differences between OLP and OHP, neither at whole-brain level nor within the different ROIs. Increased linear trend related activation in younger compared to older adults was not found either, neither at whole-brain level nor within the different ROIs.

#### Quadratic Trend

For quadratic trend related neural activation, whole-brain analyses did not reveal significant effects in none of the experimental groups.

**FIGURE 4:**
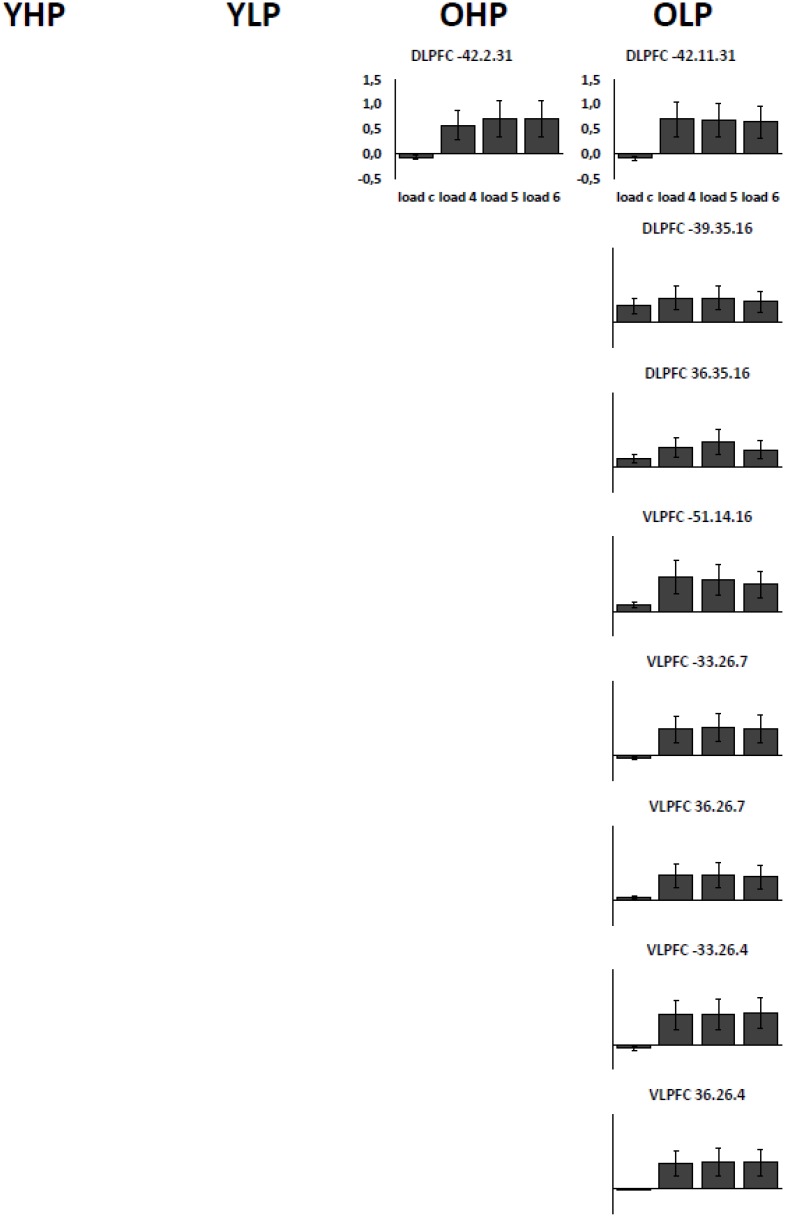
Contrast estimates with the respective standard errors of the mean for the identified regions associated with a quadratic trend. Contrast estimates are plotted for OHP and OLP for all load levels separately. YHP and YLP did not show load-related activation patterns following a quadratic trend.

**FIGURE 5:**
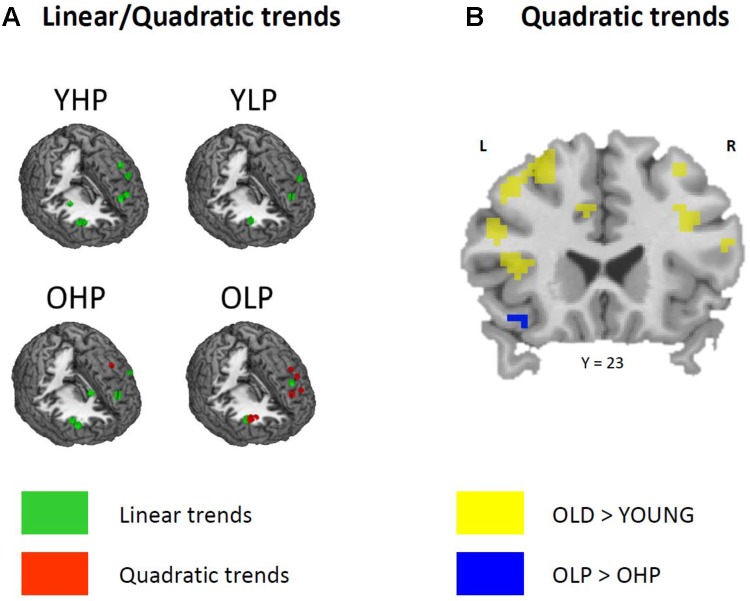
**(A)** Linear (green) and quadratic (red) trend related brain activation in different dorsolateral and ventrolateral prefrontal sub regions for YHP, YLP, OHP, and OLP. **(B)** Higher quadratic trend related activation in older compared to younger adults (yellow) and in OLP compared to OHP (blue).

Region of interest analyses showed that only older adults showed quadratic trend related brain activation in different PFC sub regions at *p* < 0.05. These trends probably indicate a dysfunctional upregulation of neural activity with increasing task load. Whereas OHP showed a quadratic trend pattern in BA 9 of the left dorsolateral PFC (see **Table 2** and **Figure 4**), OLP showed various quadratic patterns in multiple dorsolateral and ventrolateral PFC sub regions (left BA 9, bilateral BA 46, left BA 44, bilateral BAs 45 and 47). A comparison of the different trends in the four experimental groups is illustrated in **Figure 5A**.

At whole-brain level (see **Appendix**, **Table A2**), direct group comparisons between older and younger adults revealed that older adults showed stronger quadratic trend related activity than younger adults within different anterior and posterior brain regions (left middle frontal gyrus, left cingulum, right precuneus, and left middle occipital cortex). ROI analyses additionally showed increased quadratic trend related activation in older compared to younger adults (**Table 3** and **Figure 5B**) within several dorsolateral and ventrolateral PFC sub regions (bilateral BA 9, left BA 46, left BA 44, bilateral BA 45 and left 47).

**Table 3:** Increased quadratic trend activation in older compared to younger adults and in older low performers (OLP) compared to older high performers (OHP) within different dorsolateral and ventrolateral PFC sub regions (ROI analyses).

Contrast	Region	ROI	Hemisphere	*x*	*y*	*z*	*T*	*p_corr_*
OA > YA	DLPFC	BA 9	Left	-48	17	40	4.14	0.008
			Left	-3	38	37	3.82	0.022
			Left	-42	23	40	3.67	0.034
			Left	-39	2	31	3.59	0.044
			Right	3	41	37	4.36	0.004
		BA 46	Left	-51	23	25	3.56	0.016
			Left	-48	20	28	3.36	0.030
	VLPFC	BA 44	Left	-51	14	16	3.76	0.005
		BA 45	Left	-36	26	7	3.93	0.005
			Left	-48	17	16	3.83	0.006
			Left	-51	23	22	3.44	0.021
			Right	54	20	16	3.23	0.039
		BA 47	Left	-36	26	4	3.50	0.038
OLP > OHP	VLPFC	BA 47	Left	-39	26	-17	3.91	0.010

*Threshold of *p*_*corr*_ < 0.05 (FWE-corrected according to SPM12, small volume correction). All coordinates (x, y, z) are given in MNI space. DLPFC, dorsolateral prefrontal cortex; VLPFC, ventrolateral prefrontal cortex; ROI, region of interest; BA, Brodmann area.*

Corresponding differences between OHP and OLP did not reach statistical significance at whole-brain level. ROI analyses, however, indicated stronger quadratic trend related activity in OLP compared to OHP within left BA47 of the ventrolateral PFC (**Table 3** and **Figure 5B**).

Exploratory analyses for the opposite contrasts did not reveal significant effects in prefrontal brain regions.

#### Quadratic Trend × Performance Accuracy

Finally, the extent of the quadratic trend was associated with lower CBT performance accuracy in OLP, but not in OHP: Activation intensity in two of the respective peaks was positively correlated with the total number of CBT errors. Both of these peaks were located in the left dorsolateral PFC (Peak 1: MNI-coordinates -39 35 16, *r* = 0.540, *p* = 0.021; Peak 2: MNI-coordinates -42 11 31, *r* = 0.485, *p* = 0.041).

### Structural Brain Data

Whole-brain analysis revealed less GM volume in older compared to younger adults in various brain regions including different parts of the PFC (see **Appendix**, **Table A3**).

Comparing OHP with OLP, whole-brain analyses yielded no significant differences. ROI analyses, however, indicated less GM volume in OLP than in OHP within left BA 46 of the dorsolateral PFC (MNI coordinates -48 39 27, *T* = 4.26, *p* = 0.011).

### Relationship Between Functional and Structural Brain Data

Regression analyses did not reveal negative correlations between prefrontal GM volume and the extent of the positive linear trend for older adults. By contrast, we found negative correlations between GM volume of different dorsolateral and ventrolateral PFC sub regions and the extent of the quadratic trend for older adults, as well as for OLP and OHP separately (**Table 4**).

**Table 4:** Negative correlations between prefrontal GM volume and the extent of the quadratic trend in older adults (OA), older high-performers (OHP), and older low-performers (OLP).

Group	PFC sub-region	ROI	Hemisphere	*x*	*y*	*z*	*T*	*p_corr_*
OA	VLPFC	BA 44	Left	-48	17	13	4.16	0.003
		BA 45	Left	-54	17	4	4.12	0.001
		BA 47	Left	-48	35	-2	4.34	0.003
OHP	DLPFC	BA 9	Left	-45	2	28	4.36	0.041
	VLPFC	BA 44	Left	-54	8	13	4.22	0.010
			Right	54	2	22	4.27	0.010
		BA 47	Right	51	35	-2	5.13	0.007
OLP	VLPFC	BA 45	Left	-51	26	4	3.55	0.027


*Results of the ROI analyses are shown. Threshold of *p*_*corr*_ < 0.05 (FWE-corrected according to SPM12. All coordinates (x, y, z) are given in MNI space. DLPFC, dorsolateral prefrontal cortex; VLPFC, ventrolateral prefrontal cortex; ROI, region of interest; BA, Brodmann area; GM, gray matter*.

## Discussion

The results of the current study suggest that age and performance level modulate load-related neural activation in prefrontal parts of the spatial WM network. All experimental groups showed an upregulation of several dorsolateral and ventrolateral prefrontal sub regions with increasing task load (linear trends) indicating an efficient recruitment of neural resources as response to increasing WM demands, irrespective of age and performance level. Older individuals, however, additionally showed a pattern of increased activation at medium compared to low task load but stable or decreased activation at high compared to medium task load in other prefrontal sub regions as being indicated by quadratic trends. Older low-performing subjects showed this response pattern in multiple dorsolateral and ventrolateral prefrontal sub regions. The extent of this pattern was associated with reduced performance accuracy and lower prefrontal gray matter volume suggesting a regional limitation of neural resources being associated with structural deficits. The present results point toward age-related prefrontal cortex atrophy associated with possibly dysfunctional activation patterns. This reduction may first involve some dysfunctional clusters while others are unimpaired.

### Behavioral Data

In line with previous findings (Nagel et al., 2009; Toepper et al., 2010b, 2014), behavioral data analysis revealed an increasing number of errors with increasing load across all participants. The different experimental groups showed accuracy differences across all load levels with younger high-performers showing the best and older low-performers showing the poorest WM performances. Moreover, analyses revealed a significant group × load interaction indicating that the increase of task load differentially affected the increase of errors in the different experimental groups. Interestingly, older high-performers showed lower error rates than younger low-performers, possibly indicating a successful compensation for higher age.

### Neural Activation

Functional imaging data analyses revealed neural activation changes associated with increasing WM load in all experimental groups. Younger high-performers, younger low-performers, older high-performers and older low-performers all showed load effects in different prefrontal parts of the WM network. Indeed, all experimental groups showed an upregulation (positive linear trends) of several dorsolateral and ventrolateral prefrontal regions, indicating an efficient recruitment of neural resources as response to increasing task load. Contrary to our hypotheses, however, there were no group differences in the extent of neural upregulation, neither between younger and older adults nor between high- and low-performing seniors.

Importantly, only older individuals additionally showed quadratic trend patterns over the statistical threshold (i.e., increasing activation from low to medium task load but stable or decreasing activation from medium to high task load) which may reflect a recruitment of neural resources at lower task demands but exhausted neural resources at higher demand levels. In line with these findings, older individuals showed more quadratic trend related activation than younger individuals in multiple sub regions of the prefrontal cortex.

Whereas older high-performers showed this pattern in one cluster located in left dorsolateral prefrontal cortex, these patterns were more frequent in older low-performers (Nagel et al., 2009) affecting multiple prefrontal sub regions. As expected, the direct statistical comparison between high- and low-performing seniors revealed increased quadratic trend related prefrontal cortex activation in the latter group as well as an association between activation intensity and reduced performance accuracy.

Taken together, the results suggest specific prefrontal functional alterations with advanced age. Younger individuals show an efficient upregulation of prefrontal cortex activation to meet the requirements of increasing task load. Older individuals show similar patterns in several prefrontal sub regions but, in contrast to younger individuals, additional possibly dysfunctional patterns in other prefrontal cell clusters. Number and extent of these patterns appear to be lower in high-performing than in low-performing seniors, probably indicating a more preserved prefrontal functional integrity in the former group.

### Gray Matter Volume

As expected, VBM analyses revealed reduced gray matter volume of multiple brain regions in older compared to younger individuals including pronounced differences in frontal areas. These findings are in line with previous research indicating age-related regional cortical atrophy and a specific structural vulnerability of the prefrontal cortex (Raz et al., 1997; Fjell et al., 2009). Moreover, older low-performers showed lower regional brain volume than older high-performers in the prefrontal cortex (left BA 46) confirming the relevance of this brain region for cognitive performance (Kaup et al., 2011).

### Association Between Neural Activation and Gray Matter Volume

Most importantly, the results of the current work revealed a direct association between neural activation and structural integrity in older adults. As mentioned above, older adults – particularly older low-performers – showed possibly dysfunctional neural response patterns in some prefrontal sub regions as being indicated by quadratic trends. These response patterns were characterized by an increase of prefrontal activation from low to medium task load but no further increase or even a decrease of activation from medium to high task load. Our findings show that the extent of this pattern was inversely associated with regional dorsolateral and ventrolateral prefrontal gray matter volume among older individuals indicating that the dysfunctional recruitment of resources at increasing task load in older adults is related to reduced gray matter volume in the respective brain regions. Noteworthy, this structure-function relationship was found for both low- and high-performing individuals, indicating a general association between prefrontal cortical atrophy and neural prefrontal dysfunctions at higher ages. Older high-performers, however, showed the described dysfunctional neural response pattern in one cluster of the prefrontal cortex whereas this pattern was found in multiple prefrontal sub regions in older low-performers which may point toward a more preserved functional cerebral status in high-performing seniors. Confirming this assumption, quadratic trend associated activation was lower in older high-performers than in older low-performers and negatively correlated with performance accuracy only in the latter group.

## Conclusion and Limitations

In conclusion, the current work provides important information about the association between age, performance level, WM load-related neural activation and gray matter integrity. Our results suggest an upregulation of prefrontal cortex activation in response to increasing WM demands in high- and low-performing younger and older individuals as reflected by increasing activation in multiple sub regions of the prefrontal cortex. Whereas results yielded no differences between the sub groups regarding this upregulation, older individuals additionally showed dysfunctional neural response patterns in other prefrontal regions, which may reflect a recruitment of neural resources at lower task demands but exhausted neural resources at higher demand levels (i.e., increasing activation from low to medium task load but stable or decreasing activation from medium to high task load). In older low-performers, these patterns were associated with both reduced performance accuracy and gray matter atrophy in different sub regions of the prefrontal cortex probably reflecting a regional limitation of neural resources associated with prefrontal structural atrophy. In older high-performers, number and extent of dysfunctional clusters was lower than in older low-performers indicating that the cognitive status of a person does not only seem to be determined by age but particularly by the functional status of the brain. In fact, the current findings support the idea of higher brain reserve in high- compared to low-performing seniors. Brain reserve is defined as an increased adaptive neuroplasticity (Freret et al., 2015) due to less impaired prefrontal hubs and enhanced prefrontal functional connectivity (Franzmeier et al., 2017a,b). Greater brain reserve may facilitate a dynamic adjustment of neural circuits to various stressors that are associated with accelerated aging (Freret et al., 2015). In this context, a stimulating lifestyle is discussed as being a neuroprotective key factor against age-related cognitive decline (Gelfo et al., 2018).

Noteworthy, older high- and low-performers differed with respect to mean age, although age was controlled in most analyses. However, even if age differences should have attenuated some of the described effects, this only confirms that higher age is associated with increased cognitive as well as functional and structural cerebral dysfunctions. Either way, the interpretation of results does not seem to be sophisticated. Future research endeavors should focus on a further connection between cognitive, functional and structural data. The association between the current results and white matter integrity, for example, might be of particular interest. In fact, some studies revealed that the structural integrity of frontal fiber tracts was related to prefrontal cortex activation and cognitive performance (Persson et al., 2006; Schulze et al., 2011). Only the methodical combination of MRI-, DTI- and behavioral measures may allow more specific conclusions regarding the mechanisms underlying age-related cognitive decline (Minati et al., 2007; Bennett and Rypma, 2013).

## Author Contributions

All authors listed have made a substantial, direct and intellectual contribution to the work, and approved it for publication.

## Conflict of Interest Statement

The authors declare that the research was conducted in the absence of any commercial or financial relationships that could be construed as a potential conflict of interest.

## Supplementary Material

The Supplementary Material for this article can be found online at: https://www.frontiersin.org/articles/10.3389/fnagi.2018.00265/full#supplementary-material

## Footnotes

1 http://www.fil.ion.ucl.ac.uk/spm/

2 http://www.neuro.uni-jena.de/vbm

3 http://www.fil.ion.ucl.ac.uk/spm/

4 www.brain-development.org
