# Supplementary material for: Performance Level and Cortical Atrophy Modulate the Neural Response to Increasing Working Memory Load in Younger and Older Adults
Source: Front Aging Neurosci. 2018 Sep 11;10:265. doi: 10.3389/fnagi.2018.00265 (PMC6141635; doi:10.3389/fnagi.2018.00265)
Supplement: Supplementary file 1 [file Data_Sheet_1.docx]

**Appendix**

**Table A1**. Positive linear and quadratic trend related whole-brain activation, separately for younger high-performers (YHP) and younger low-performers (YLP).

| **trend** | **group** | **region** | **hemisphere** | ***x*** | ***y*** | ***z*** | ***T*** | ***p_corr_*** |
| --- | --- | --- | --- | --- | --- | --- | --- | --- |
| Linear | YHP | Parietal inferior | Left | -36 | -49 | 49 | 8.31 | .004 |
|  |  | Frontal superior | Right | 30 | 11 | 61 | 8.04 | .006 |
|  |  | Frontal superior | Left | -27 | 5 | 67 | 7.98 | .007 |
|  | YLP | Frontal superior | Left | -21 | 2 | 55 | 9.49 | .002 |
|  |  | Frontal superior | Right | 27 | 5 | 61 | 8.42 | .005 |

*Note:* Threshold of *p*_corr_ < 0.05 (FWE-corrected according to SPM12, minimum cluster size = 10). All coordinates (x, y, z) are given in MNI space.

**Table A2**. Increased quadratic trend related whole-brain activation in older compared to younger adults.

| **region** | **hemisphere** | ***x*** | ***y*** | ***z*** | ***T*** | ***p_corr_*** |
| --- | --- | --- | --- | --- | --- | --- |
| Precuneus | Right | 15 | -52 | 19 | 5.10 | .016 |
| Cingulum middle | Left | -6 | -40 | 37 | 5.04 | .019 |
| Cingulum posterior | Left | -6 | -43 | 13 | 5.01 | .021 |
| Frontal middle | Left | -33 | 14 | 52 | 4.95 | .025 |
| Occipital middle | Left | -39 | -76 | 37 | 4.92 | .028 |

*Note:* Threshold of *p*_corr_ < 0.05 (FWE-corrected according to SPM12). All coordinates (x, y, z) are given in MNI space.

**Table A3**. Decreased grey matter volume in older compared to younger adults.

| **Brain structure** | **hemisphere** | ***x*** | ***y*** | ***z*** | ***T*** | ***p_corr_*** |
| --- | --- | --- | --- | --- | --- | --- |
| Cerebellum | Right | 29 | -77 | -39 | 7.32 | 0.000 |
| Postcentral Gyrus | Left | -44 | -21 | 42 | 6.82 | 0.000 |
| Inferior lateral parietal lobe / postcentral gyrus | Left | -60 | -23 | 42 | 6.62 | 0.001 |
| Cerebral White Matter | Right | 3 | 2 | -2 | 6.54 | 0.001 |
| Inferior frontal gyrus | Left | -47 | 30 | 12 | 6.52 | 0.001 |
| Cerebellum | Left | -32 | -72 | -41 | 6.48 | 0.001 |
| Inferior frontal gyrus / insula | Right | 38 | 23 | -3 | 6.39 | 0.002 |
| Superior frontal gyrus | Right | 17 | 27 | 39 | 6.24 | 0.003 |
| Superior frontal gyrus | Right | 17 | 41 | 35 | 6.24 | 0.003 |
| Superior parietal gyrus | Right | 2 | -45 | 59 | 6.09 | 0.005 |
| Superior frontal gyrus | Left | -24 | 27 | 53 | 6.06 | 0.005 |
| Superior frontal gyrus | Left | -18 | 29 | 35 | 6.04 | 0.006 |
| Middle frontal gyrus | Left | -47 | 9 | 36 | 6.04 | 0.006 |
| Postcentral gyrus | Right | 62 | -15 | 14 | 6.00 | 0.007 |
| Precentral gyrus | Left | -33 | -21 | 57 | 5.94 | 0.008 |
| Postcenral gyrus | Right | 42 | -21 | 47 | 5.76 | 0.015 |
| Precentral gyrus | Left | -27 | -2 | 15 | 5.75 | 0.016 |
| Postcentral gyrus | Right | 59 | -18 | 42 | 5.74 | 0.016 |
| Cerebellum | Left | -24 | -45 | -41 | 5.73 | 0.017 |
| Cerebellum | Left | -45 | -53 | -45 | 5.73 | 0.017 |
| Precentral / postcentral / superior temporal gyrus | Right | 60 | -2 | 6 | 5.72 | 0.017 |
| caudate nucleus | Left | -18 | 5 | 9 | 5.62 | 0.024 |
| posterior temporal lobe | Right | 63 | -41 | 2 | 5.57 | 0.028 |
| postcenral/ precentral gyrus | Right | 30 | -26 | 59 | 5.55 | 0.030 |
| Superior frontal gyrus | Left | -11 | 24 | 51 | 5.53 | 0.033 |
| Inferior / middle frontal gyrus | Right | 50 | 42 | -5 | 5.45 | 0.042 |

*Note:* Threshold of *p*_corr_ < 0.05 (FWE-corrected according to SPM12). All coordinates (x, y, z) are given in MNI space.
